# Surgical treatment in Osteogenesis Imperfecta – 10 years experience

**Published:** 2013-06-25

**Authors:** I Georgescu, C Vlad, TȘ Gavriliu, S Dan, AA Pârvan

**Affiliations:** “Maria Sklodowska Curie" Clinical Emergency Hospital for Children, Bucharest

**Keywords:** osteogenesis imperfecta, fractures, coxa vara, pseudarthrosis, bone grafts

## Abstract

Introduction. Osteogenesis imperfecta (OI) is a very rare disease compared to other afflictions, running the risk of social isolation for children and their parents, due to the problems specific to the disease. All the social, psychological and physical disadvantages must be removed or at least mitigated, all within the society’s limited resources.

In Romania, this situation has led in the last couple of years to the selection of a number of extremely severe cases, which could not be solved by orthopedic and classic surgical treatment methods. These patients exhibit gracile long bones, which are distorted, often with cystic degeneration at the level of the extremities, pseudarthroses, limb length discrepancies, most of them being unable to walk, being condemned to sitting in a wheelchair.

Aim. This paper deals with the experience of the Orthopedics Department of "Maria Sklodowska Curie" Clinical Emergency Hospital for Children, in Bucharest, in the field of surgical treatment for moderate and severe forms of OI, within the time frame of May 2002-May 2012. For the first time in Romania, on May 20, 2002, the team led by Professor Gh. Burnei, MD, has implanted telescopic rods in the femur and tibia of a patient with OI.

One of the most important themes, of great interest in the orthopedic surgery, is the osteoarticular regularization and reconstruction in severe forms of OI, which should allow the patients to stand and walk. These cases are a challenge for the surgeon, who is in the position of applying new, complex procedures, or perfecting, modifying and adapting techniques that have already been established. The aim of the surgical treatment is the increase of the quality of life of these children and adolescents and of their social integration.

Methods and results. In the above-mentioned period, from the OI patients who are in the evidence of our clinic, 32 were operated on, totaling 81 surgeries. Out of these, 28 patients, aged 2-27 years, have benefited from reconstructive surgery of the pelvic limbs. Sofield-Millar osteotomies were practiced and 69 Sheffield telescopic rods were implanted in 25 patients and 43 surgeries. The coxa vara / valga correction using the Sheffield rod was applied in 6 patients and 8 hips, respectively. Circular or monoplane external fixators were used in 7 patients for the correction of deformities, lengthening and arthrodiastasis. 9 patients have benefited from various forms of bone transplant: pedicled grafts, auto- and/or allografts. An original bone reconstruction procedure is currently being studied and will be useful in the treatment of large bone defects and the thickening of the gracile diaphyses, which consists in practice of a massive contribution of free bone grafts, auto- and/or allogenic, bone substitutes and, in selected cases, periosteal substitutes, in a composite stratified construction.

Postoperatively, 15 patients are able to walk while being supported by crutches or walking frames, 5 patients walk independently and 8 are still wheelchair-bound. It is important to mention that 8 children who were preoperatively dependant on the wheelchair are now walking!

Discussion. The surgical treatment in severe forms of OI must be adapted to each case. No matter the surgical technique used, well known or innovative, it is convenient if it restores the ability to walk of a youngster who has been forced to use a wheelchair for almost 20 years and who has suffered dozens of unsuccessful surgeries. The current paper mainly describes the difficulties the surgeon has to deal with while treating the severe, neglected cases of OI, sometimes incorrectly cared for and labeled as inoperable.

## Introduction

It is unanimously accepted that the defining element for the osteogenesis imperfecta (OI) is the fragility of bone [**1**]. OI has also been defined by the clinical triad “fragile bones, blue sclerae, early deafness" [**2**].

The disease is genetic: the genetic defect is either inherited, with autosomal dominant or recessive transmission, or it appears as a result of spontaneous mutations. 150 mutations have been described at the level of COL 1 A1 and COL 1 A2 genes, which induce quantitative and qualitative disorders of type I procollagen synthesis [**3**]. 

 From the anatomical-pathological point of view, the structure of the tissues and the organs that contain type I collagen will be altered: bones, the ossicles of the internal ear, teeth, sclerae, skin, blood vessels, capillaries, and ligaments. 

 The clinical picture is dominated by the musculoskeletal manifestations: frequent fractures, bowing of the long bones, craniofacial dysmorphism, ligamentous and articular hyperlaxity, muscular hypotonia. The fractures generate bowing and, in a vicious circle, the bowing favors the appearance of fractures. The number of fractures significantly decreases beyond the age of adolescence. The extraskeletal manifestations can be of the following types: dentinogenesis imperfecta, deafness, blue sclerae, capillary fragility, sweating, tachycardia, tachypnea, etc. [**4**].

**Fig. 1:**
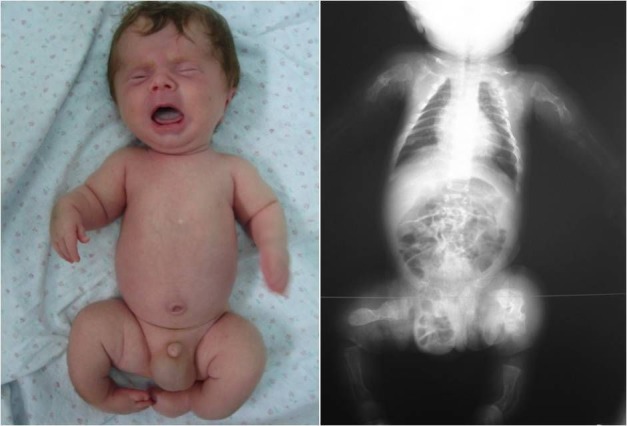
Congenita A type of osteogenesis imperfecta in a 2-month-old child

 This affection is difficult to define and classify due to the wide spectrum of manifestations, from lethal forms in the perinatal period to the subtle forms, which can be hard to identify, compatible with a normal life. Shapiro´s (**Table 1**, **Fig. 1**) [**5**] and Sillence´s classifications are based on clinical criteria. To the 4 types initially described by Sillence [**6**], types V and VI have been added by Glorieux [7,8] and type VII by Ward (**Table 2**) [**9**]. Sillence himself, whose classification remains the most used of all, suggested the existence of more than 12 types of OI [**2**].

**Table 1:** Shapiro Classification

Type	Clinical
Congenita A	In utero fractures / at birth, fragmented femurs, 94% mortality rate
Congenita B	In utero fractures/ at birth, normally contoured bones, 8% mortality rate, 59% dependant on the wheelchair, 33% able to walk
Tarda A	Fractures before walking age, 33% dependant on the wheelchair, 67% being able to walk
Tarda B	Fractures after the walking age, 100% being able to walk

**Table 2:** Sillence classification, modified by Glorieux and Ward

Type	Genetics	Severity	Teeth	Sclerae	Other characteristics
I	Autos. dom.	Light form	Normal	Blue	
II	Autos. rec.	Lethal form	Dentinogenesis imperfecta	Blue	
III	Autos. rec. / dom.	Severe form	Dentinogenesis imperfecta	Bluish	
IV	Autos. dom.	Moderate form	Dentinogenesis imperfecta or normal	White	
V	Autos. dom.	Moderate form	Normal	White	Hypertrophic calluses Limited pronosupination of the forearms (calcification of the interosseous membrane)
VI	Probably autos. dom.	Extremely rare moderate form	Dentinogenesis imperfecta or normal	White	Vertebral fractures Elevated alkaline phosphatase
VII	Autos. rec.	From a moderate to extreme	Dentinogenesis imperfecta or normal	White / blue	Rhizomelic form (disharmonic pelvic and thoracic limbs, due to involvement of the femur and humerus) Dwarfism Coxa vara

 Genetic counseling is important for the families with members affected by OI, who wish to have children. The prenatal diagnosis of the severe forms is possible by fetal ultrasound, starting with the14th-18th week of pregnancy. The less severe forms can easily be overlooked during the ultrasound diagnosis [**10**]. During the first trimester of pregnancy, the biopsy of the chorionic villosities, genetic and molecular biology tests can establish the diagnosis of OI, giving the parents the option of terminating the pregnancy if severe forms are discovered [**11**]. 

 The medication in OI consists in the administration of bisphosphonates, the age of administration being below 2 years old; however, it is continually decreasing. Their role is substantial: they decrease the frequency of fractures, increase the bone density and improve the quality of life, due to the positive effect on the ambulatory status of the patient [**12**]. The monitoring of the bone density by osteodensitometry (ODM) is mandatory and it is done annually. There are studies that have demonstrated that there is a limit to the efficiency of bisphosphonates, beyond which they do not bring any further improvement, only adverse effects [**13**]. Because of this, the administration of bisphosphonates for more than 2 years is usually not justified. The doses are adjusted according to the age and weight. 

 A fundamental principle in OI treatment is the encouragement of physical activity. 

 Orthopedic treatment, consisting of plaster immobilizations, can be useful, though the period of immobilization must be reduced as much as possible, because it accentuates osteoporosis, muscular hypotonia and favors the onset of pseudarthrosis. In fact, the curved long bones should be braced intramedullarily with telescopic rods, before the appearance of fractures. The moderate forms can be treated this way, in an almost minimally invasive manner. 

**Fig. 2:**
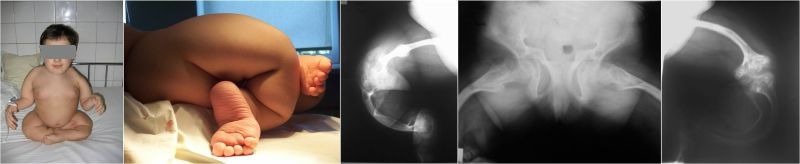
A 13-year-old patient, displaying an extremely severe form of OI, with monstrous deformities of the pelvic limbs, who has never walked. For each pelvic limb, monoplane external fixators have been used in order to elongate the soft tissues and the bone, thicken the diaphysis by lateral traction on olive wires and realign the segments. All 4 segments have been splinted with Sheffield rods and rib allografts were needed around the extremely thin diaphysis. The reconstructive surgery has restored orthostatic position with the help of bilateral knee-ankle-foot orthotics.

 Severe cases of OI impose the use of new complex surgical techniques, some of them innovative, by which the thickening of the gracile diaphyses can be obtained, along with the correction of the severe distortions and bowing, simultaneous correction of the coxa vara deformities, rarely of coxa valga, treatment of pseudarthroses, adaptation of the implants to the cystic structure of the metaphyses of the long bones, equalization of the pelvic limbs. Usually, these forms are incompatible with walking (**Fig. 2**). 

 The severe forms are the result of the following: (a) patients with lethal risk, type II OI, who miraculously survive; (b) neglected cases of type I, III-VII OI, with malunited fractures, distortions and bowing of the bones; (c) some cases operated in a classic fashion, for example with intramedullary Kuntscher or Rush rods, which postoperatively exhibit, at distance, straight femoral diaphyses, but with severely deformed metaphyseal-diaphyseal extremities (coxa vara / valga, femur distortions, deformations of the femoral condyles); (d) treatment errors, like prolonged plaster immobilizations, excessive and unjustified limitations of the physical activity, the bracing of long bones with classical implants in a growing child. 

 The surgical techniques for regularization and osteoarticular reconstruction of the pelvic limbs in the severe forms of OI have as their main purpose the improvement of patient comfort, the reduction of physical disability and, why not, the restoration of the ability to stand and walk. 

## Methods

The present paper is based on a descriptive study, which analyses the surgically treated cases in our clinic, between 2002 and 2012, focused on the severe forms that have always constituted a challenge for the orthopedic surgeon. The data in literature have been confronted with the data resulted from the 10 years study, reported in the surgically treated severe cases of OI.

46 patients with different forms of OI, severe, moderate or simple were in the evidence of our clinic during this period. The patients with severe and moderate forms had treatment regimens, which implied bisphosphonates therapy, neridronate and, starting with 2010, pamidronate – parenteral administration, repeated every 4 months, while monitoring urea, creatinine, serum calcium, phosphatemia and alkaline phosphatase. ODM, which was repeated annually, has shown the increase of bone density in almost every case. The treatment was usually stopped after 2 years, the maximum period of administration being 5 years. The scheme of treatment for pamidronate varied according to the age: below 2 years – 0.5 mg/kg/day; between 2 and 3 years – 0.75 mg/kg/day; over 3 years – 1 mg/kg/day. The youngest patient who has benefited from a treatment with pamidronate was 8 months old. Bisphosphonate treatment was stopped 4-6 months preoperatively and 2 – 4 months postoperatively for the operated patients. Some studies have shown that bisphosphonates delay the consolidation of the osteotomy, but not of the fractures [**14**].

 During a period of 10 years, 32 patients with OI were operated (81 surgeries), out of which 28 had moderate and severe forms and needed osteoarticular reconstructive surgery techniques of the pelvic limbs. The patients’ age ranged 2 to 27 years old. 

 Intramedullary telescopic rodding 

 Surgical treatment for OI is based on the Sofield-Millar technique, described in 1959, which consists of multiple osteotomies of the bowed long bones followed by intramedullary rodding using, at that time, Kuntscher or Rush rods. Although they ensure straight diaphyses, the classic rods do not control the severe deformities of the long bone extremities. On the other hand, the implant remained short as the bone continued to grow in length, also adding the risk of growth plate lesions.

 These disadvantages have led to the appearance of the first telescopic rods, the Bailey-Dubow rods with detachable extremities in the form of the letter “T" (1963). Sheffield rods feature fixed heads in the form of the letter “T", but in order to be implanted the opening of the joints adjacent to the splinted bone segment is necessary. Fassier-Duvall rods have been designed so that the fixation is done by screwing them in the epiphyses, without opening the joints. The possible complications after telescopic rodding are the following: migration, lack of telescoping, fractures on the rod, lesions of the growth plates, bending of the rod, rotation disorders of the operated segment, infections [**4,15**].

The ambulatory status of the patients improves after the telescopic rod surgery, especially when it is associated with the intravenous administration of bisphosphonates [**16**]. 

 For the first time in Romania, the surgical team led by Prof. Gh. Burnei, MD, used telescopic rods in a patient with OI, operated on May 20, 2002. Since then, 25 patients have benefited from 43 similar surgeries, out of which, 27 surgeries were done for both the femur and tibia on the same side. A total amount of 69 Sheffield rods was implanted. Although the telescoping is useful for the growing bones, we must admit that we have used Sheffield rods also in the patients who were not in the growing stage anymore, due to the lack of a better implant which could be fixed in some degenerate cystic bone extremities, with a “popcorn" aspect on the X-ray images. 

 Coxa vara / valga correction using the Sheffield rod 

 Coxa vara has a high prevalence in patients with severe forms of OI (especially type III), reaching 10,2%. In OI, the bowing of the femur’s proximal diaphysis can be wrongly interpreted as coxa vara. The femoral diaphyseal-cervical angle is measured and, if the resulting value is less than 110 degrees, coxa vara is diagnosed. This deformity increases the functional deficits caused by bone fragility and the preexisting deformities [**17**].

**Fig. 3:**
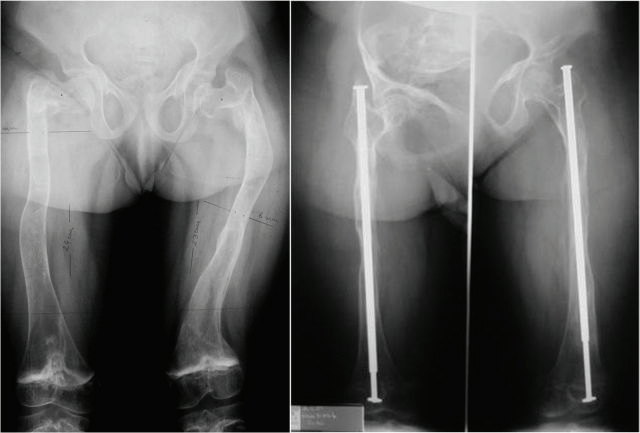
Coxa vara correction on Sheffield rod

 The surgical straightening is useful and we have resorted to correction using the Sheffield rod. The procedure consists of subtrochanteric osteotomy and the rod´s insertion through the piriform fossa and the lateral cortex in the intertrochanteric area [**4**]. Using this technique, 6 hips exhibiting coxa vara were surgically corrected in 4 patients (**Fig. 3**). Correction of coxa valga using a telescopic rod was used in 2 patients, for 2 hips. 

 The use of external fixators in the treatment of OI 

 The application of external fixators on the extremely fragile bones represents an act of courage. The first bone lengthening in adult patients with OI has been described by Ring and col., in 1996 [**18**]. Using Ilizarov frames, the lengthening of the segment averaged 6.6 cm. In 2004, Saldanha and col. reported a series of 6 children, who underwent limb lengthening ranging from 3.8 to 8.5 cm per bone segment [**19**].

 All the authors agree that the best time for lengthening, in order to equalize the lower limbs, is after the end of growth, especially when the length discrepancy exceeds 5 cm. The healing index is comparable to the one in the lengthening of a healthy bone. The complications, when they do appear, are related to the type of frame used and not to bone dysplasia [**18**].

 The limb length discrepancies in OI patients can be: (a) relative – caused by the bowing, which require the lengthening of soft tissues and (b) absolute – needing bone lengthening. 

**Fig. 4:**
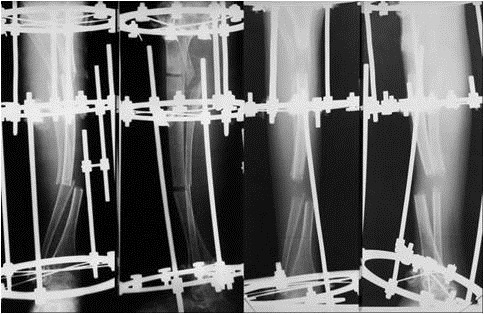
Tibial double focus lengthening - 7 cm were gained (radiological images from the process of progressive lengthening)

 In November 2002, an external fixator was used for the first time in an OI patient in our clinic, the main purpose being bone lengthening and correction of the axis of the pelvic limb. The 16-year-old patient had an Ilizarov type external fixator applied for the deflection of the right knee and an 8 cm right femur lengthening. After 18 months, a 7 cm lengthening of the right tibia, in a double focus, was practiced in the same patient (**Fig. 4**). 6 patients have benefited from external fixator lengthening (6 thighs and 4 calfs), achieving between 2 and 8 cm lengthening per bone segment and the lengthening of the soft parts. In one patient, the Ilizarov external fixator was used for the deflection of a knee. 

 Thickening of the diaphyses of the long bones and the treatment of pseudarthrosis 

 The orthopedic surgeon is forced to find treatment solutions for filiform diaphyses, frequently with bone defects like pseudarthrosis, in severe cases of OI. Although useful, the simple periosteal elevation is not enough. Lateral traction with olive wires using an external fixator for the thickening of the diaphyses has been used in one patient, for the right femur and tibia [**4**]. In severe cases, bone autografts have been used as a modality of treatment along with bone allografts and various bone substitutes, an area of great interest in pediatric orthopedics. 

 Bone transplant in OI

 The role of bone transplant in OI is to achieve a bone structure appropriately sized with age and which will subsequently integrate so that the bone resistance will offer the patient the possibility to stand and walk. The bulkier epiphyses must be preserved in order to maintain the mobility of the adjacent joints. The preservation of the growth plates is mandatory in growing children. 

 The methods for thickening the bones were the following: (a) pedicled bone grafts; (b) free, corticospongious bone grafts; (c) composite bone graft with circumferential compression by reconstruction plate, according to Burnei´s procedure [**20**].

 (a) The pedicled bone grafts can come from the following sources: 

 1. Adequate autogenic or allogenic bone tissue taken from brain-dead patients (the fibula and tibia are preferred); an anastomosis is done between the vascular pedicle of the graft and the femoral or deep femoral artery, for the thigh, or with the posterior tibial artery, for the calf; 

 2. Lateral or longitudinal bone transport, using an external fixator or by open surgery. 

**Fig. 5:**
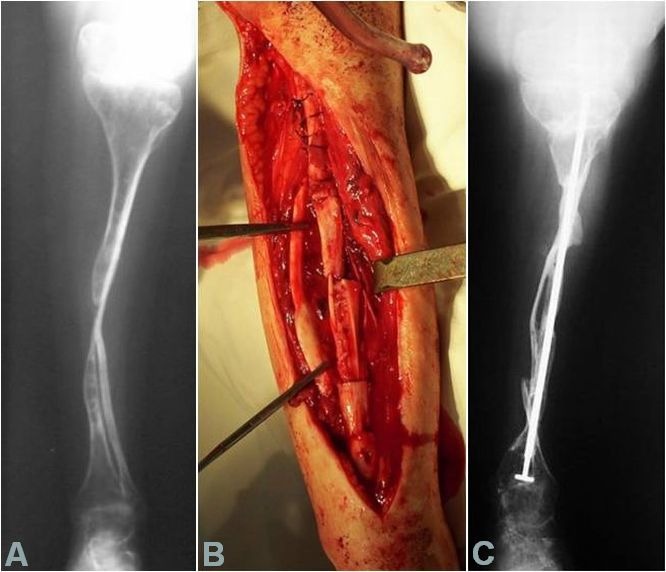
Loose tibial hypotrophic pseudarthrosis in a 21-year-old patient with OI, who has not walked since she was 2 years old. B. Fibular pedicled autogenous graft, by open lateral transport, after the surgical treatment of the pseudarthrosis using a Sheffield rod. C. Radiological aspect at 1-year postoperatively. At present, the patient walks with crutches.

 This treatment method has been applied in a single patient, known to us from the age of 21, who had approximately 22 fractures of the lower limbs, out of which 10 were operated, with loose hypotrophic pseudarthroses of both bones of the left leg, bilateral coxa valga and a 9.5 cm shortening of the left pelvic limb (**Fig. 5**). The surgeries done in our clinic on all the 4 segments of the legs have given this patient the ability to walk assisted after 19 years of using the wheelchair. 

**Fig. 6:**
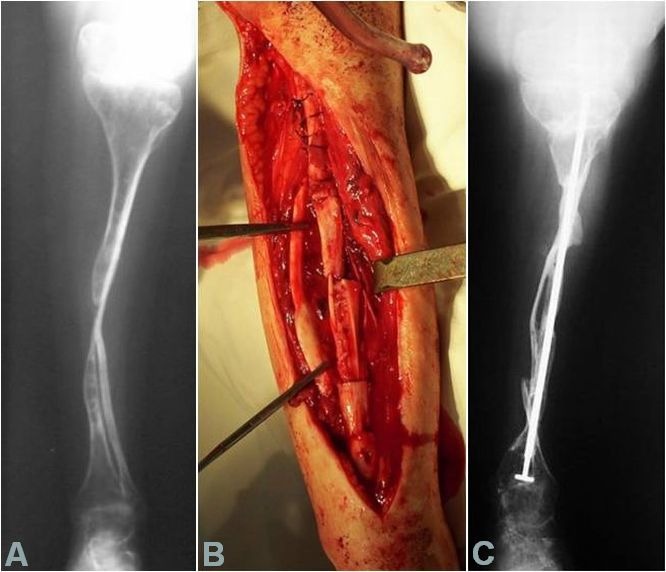
Intraoperative and postoperative radiological aspect of the rib allografts around a femoral diaphysis, connected with slowly absorbable thick sutures

 (b) There have been patients for whom the thinnest Sheffield rod (3 mm) was thicker than the diaphysis of the long bone to be splinted. However, the decision was made to implant the rod regardless. The bone fragments resulting from the lengthwise splitting of the diaphysis were fixed with thick sutures and a massive amount of rib allografts were added around and compacted with slowly absorbable sutures (**Fig. 6**). 

**Fig. 7:**
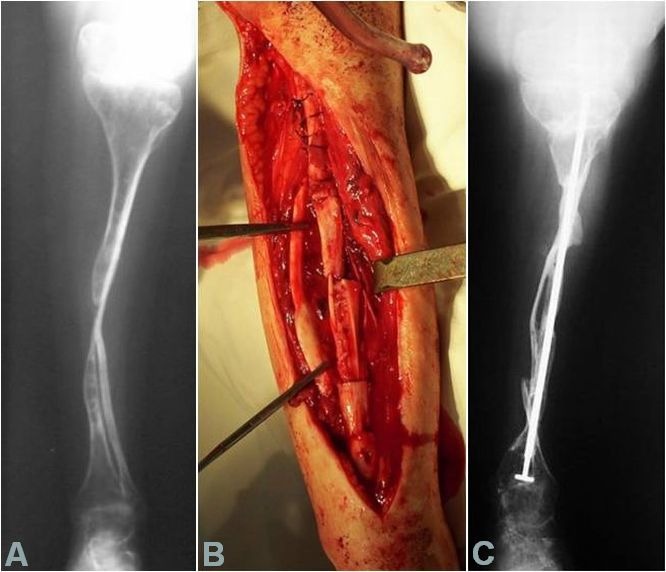
A. Double pseudarthrosis of the right femur in a 27-year-old patient, operated when she was 19 years and 10 months old, by using the Sofield-Millar technique. The Sheffield rod broke 6 years after surgery. B. Massive bone grafting of the right femur using a composite construction

 The severe forms of OI, multiply operated, in patients who cannot walk and wheelchair-bound, have oriented and stimulated the development of an original bone transplant procedure, by which a complex structure is built around the gracile diaphyses, in concentric layers [**20**]:

 1. The main axis is preserved, made up of the osteoporotic bone splinted with elastic rods (TEN), Sheffield telescopic rod or other special rods. 

 2. A compartment is formed around the main axis, which is made up of the following: 

 2.1. Rib allogenic bone grafts, each on an elastic splint (K wire or TEN), with osteoinductive properties, but mostly osteoconductive, ensuring the mechanic resistance of the assembly [**21**];

 2.2. Osteoinductive substances containing Demineralized Bone Matrix (DBM), with the aim of increasing the osteoinduction, stimulating the transformation of the recipient’s mesenchymal cells into osteoblasts [**21**];

 2.3. Periosteal grafts, in the case of pseudarthroses; 

 3. When the space allows it, solid pylons made of femoral diaphysis allograft are implanted, which are then fixed with wires or cables around a reconstruction plate in order to compact the allogenic grafts. When possible, the plate is fixed at its extremities with 1-2 screws (**Fig. 7**). 

 This bone reconstruction technique has been applied for the first time in patients with OI, subsequently being extended to the cases of congenital pseudarthroses of the tibia. There have been 3 patients with OI – 2 females and 1 male – operated in this manner on 4 segments: 2 femurs and 2 tibias. There has been a loose pseudarthrosis of the tibia and a two-level tight pseudarthrosis of the femur (**Table 3**). 

**Table 3:** Three patients with severe forms of OI, wheelchair-bound, underwent reconstructive surgery for lower limbs by massive bone grafting in a composite, stratified construction

Case	Gender	Anamnestic data	Op. segm.	Pseudarthrosis type	Surgical treatment
CM 19y	F	19y10m: SM osteotomies, Sheffield telescopic rodding for R femur and R tibia 6y later: broken rod R femur WHEELCHAIR-BOUND IN THE LAST 3 YEARS!	R femur	tight, two-level pseudarthroses	27y3m: intramedullary splint, fibular, femoral, rib allografts, with K wires, ALLOMATRIX, Dall-Miles plate
ADR 21y6m	F	14y: Rush rods for both femurs 18y: fibular allografts from the mother, fixed by plates and screws at the level of tibias UNABLE TO WALK SINCE SHE WAS 11Y6M OLD!	R tibia L femur	Loose pseudarthrosis Bowing	24y2m: implant removal, pseudarthrosis cure, fibular allograft, rib allografts splinted with K wires, Dall-Miles plate, periosteal substitute (RT) 25y5m: massive allografting using femoral diaphysis, ribs, Dall-Miles plate, ALLOMATRIX 10cc, GRAFT JACKET (LF) 26y2m: Dall-Miles plate and cables removed from the R tibia AMBULATES WITH WALKER FRAME!
IDA 2y	M	3y11m: SM osteotomies, Sheffield telescopic rodding for the R tibia 6y: SM osteotomies, Sheffield telescopic rodding for the L femur WHEELCHAIR-BOUND SINCE HE WAS 4 YEARS OLD!	R tibia	Bowing	10y1m: SM osteotomies, intramedullary synthesis with 2mm TEN, rib and femoral allografts, ALLOMATRIX, Dall-Miles plate 11 days po: tibial fracture at the distal end of the plate – closed reduction WALKS WITH SUPPORT AND ORTHOSIS!

**Fig. 8:**
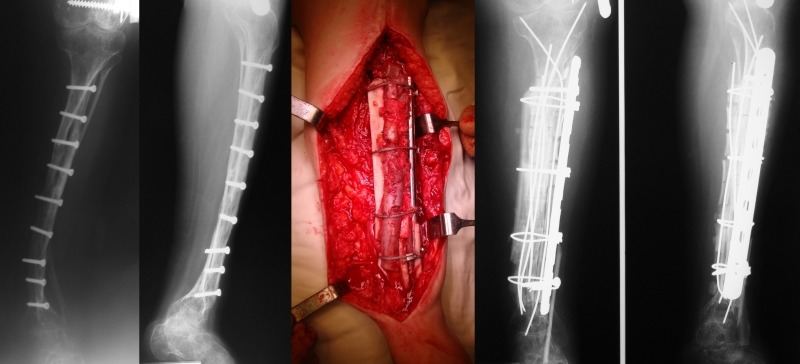
Burnei´s procedure

 After surgery for the tibia, immobilization in a cast for 6-12 weeks was needed. The first patient in the series, whom we have known since the age of 21 years and 6 months, had suffered over 20 surgeries beforehand, exhibiting a loose hypotrophic pseudarthrosis of the right tibia, being dependant on the wheelchair since she was 11 years old. She underwent 3 surgeries in our clinic, out of which the first was at the age of 24: right tibia composite transplant, left femur composite transplant, extraction of the Dall-Miles plate from the right tibia at 2 years after implantation. Currently, at 1 year from the last surgery, she is able to walk with crutches (**Fig. 8**). 

## Results

All the patients in the study had a presentation sheet, based on a rigorous anamnesis, which allowed the determination of OI type. The number of fractures, the ODM values, the values of the blood tests and all the treatments were monitored. The patients were followed up clinically and radiologically. The effects of the treatments on the ambulatory status were also analyzed. This way, after the reconstructive surgery for the lower limbs, done in 28 patients with moderate and severe forms of OI, we have noticed that 15 patients are able to walk with support (crutches or walking frames), 5 are walking independently, while 8 are still wheelchair-bound. 

**Fig. 9:**
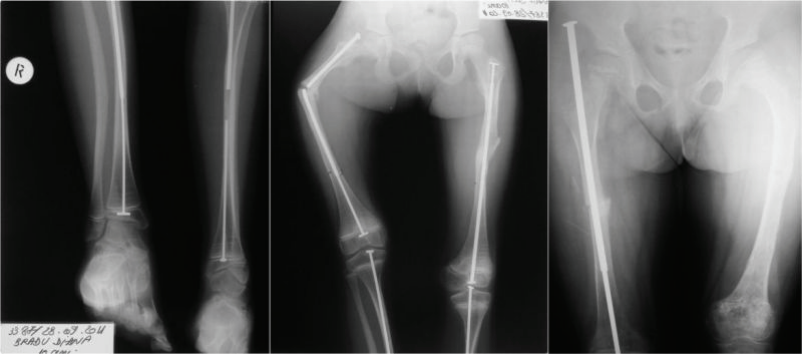
Possible complications after Sheffield telescopic rodding: bending, disengagement, fracture of the rod, migration

 Reconstructive surgery of the lower limbs does not lack complications. After the implant of the Sheffield telescopic rods we have recorded the following: 7 external migrations of the obdurate rod, 7 pseudarthroses, 3 fractures with the bending of the rod, 2 fractures with the breaking of the rod, 1 fracture on disengaged rod, 1 case of osteitis of the femur, 1 case of cellulitis, 1 tibia fracture below Dall-Miles plate with hematoma and 1 case with wound dehiscence (**Fig. 9**).

 For cases with massive bone grafting, wound care is difficult, occasionally with difficult healing because of the pressure exerted on the skin by the high volume of the fitting (allografts, bone substitutes, osteosynthesis materials). In one patient, in whom the tibia has been reconstructed using the composite model, an angular fracture of the bone at the distal end of the plate has occurred, which needed reduction and cast immobilization and, at the same time, a superficial hematoma was cared for at the level of the operated calf. 

## Discussion 

The prenatal diagnosis of the severe forms of OI allows for abortion at 12-14 weeks of pregnancy. Genetic counseling for families with affected members has a substantial role, avoiding family dramas. 

 There should not be any more patients with this affliction who reach adulthood with no other treatment except for orthoses, dependant on their family and barely integrated in society. 

 The chance of the patients with severe forms of OI resides in reconstructive surgery, a field of great interest, which is in a permanent process of transformation. Currently available implants do not always satisfy the requirements of a particular case, their better adaptation to the clinical case is necessary, or, why not, the development of new models. Intramedullary rods made of a nickel and titanium memory alloy [**22**], as well as bone marrow transplant [**21**] could represent the future in the treatment of the disabling forms of this rare disease. Implants dedicated to other pathology have proved to be life saving in some cases; we are talking here about the use of Dall-Miles plates in the massive bone transplant, according to the composite model, which was previously described, for the compaction of the bone allografts. 

 The OI patients must be enrolled in a National Register of Patients with Osteogenesis Imperfecta, in order for us to have clear records, thus avoiding the neglected evolutions and the physical and mental handicap of these patients. 

 The fact that 8 patients who were condemned to use the wheelchair preoperatively are now able to walk, some even after 20 years, is impressive. In these situations, the adopted surgical technique, dedicated or innovative, can only be advisable.

 Sources of finance

 This paper is supported by the Sectoral Operational Programme Human Resources Development (SOP HRD) 2007-2013, financed from the European Social Fund and by the Romanian Government under the contract number POSDRU/107/1.5/S/82839.

## References

[1] Zeitlin  L, Fassier  F (2003). Modern approach to children with osteogenesis imperfecta. J Pediatr Orthop B.

[2] Plotkin  H (2006). Two Questions about Osteogenesis Imperfecta. J Pediatr Orthop.

[3] Kocher  MS, Shapiro  F (1998). Osteogenesis imperfecta. J Am Acad Orthop Surg.

[4] Burnei  G, Vlad  C (2008). Osoegenesis Imperfecta: Diagnosis and Treatment. J Am Acad Orthop Surgery.

[5] Shapiro  F (1985). Consequences of an osteogenesis imperfecta diagnosis for survival and ambulation. J Pediatr Orthop.

[6] Sillence  DO, Senn  A (1979). Genetic hetrogeneity in osteogenesis imperfecta. J Med Genet.

[7] Glorieux  FH, Rauch  F (2000). Type V Osteogenesis Imperfecta: a New Form of Brittle Bone Disease. J Bone Miner Res.

[8] Glorieux  FH, Ward  LM (2002). Osteogenesis Imperfecta Type VI: a Form of Brittle Disease with a Mineralization Defect. J Bone Miner Res.

[9] Ward  LM, Rauch  F (2002). Osteogenesis imperfecta type VII: An autosomal recesssive form of brittle bone disease. Bone.

[10] Thompson  EM (1993). Non-Invasive Prenatal Diagnosis of Osteogenesis Imperfecta. Am J Med Genet.

[11] Buisson  O, Senat  MV (2002). Update on prenatal diagnosis of osteogenesis type II: An index case report diagnosed by ultrasonography in the first trimester. J Gynecol Obstet Biol Reprod.

[12] Hickey  J, Lemons  D (2006). Bisphosphonate Use in Children With Bone Disease. J Am Acad Orthop Surg.

[13] Vallo  A, Rodriguez-Leyva  F (2006). Osteogenesis imperfecta: Anthropometric, skeletal and mineral metabolic effects of long-term intravenous pamidronate therapy. Acta Paediatr.

[14] Munns  CF, Rauch  F (2004). Delayed osteotomy but not fracture healing in pediatric osteogenesis imperfecta patients receiving pamidronate. J Bone Miner Res.

[15] Mulpuri  K, Joseph  B (2000). Intramedullary rodding în osteogenesis imperfecta. J Pediatr Orthop.

[16] El-Sobky  MA, Hanna  AA (2006). Surgery versus surgery plus pamidronate in the management of osteogenesis imperfecta patients: A comparative study. J Pediatr Orthop B.

[17] Aarabi  M, Rauch  F (2006). High prevalence of coxa vara in patients with severe osteogenesis imperfecta. J Pediatr Orthop.

[18] Ring  D, Jupiter  JB (1996). Treatment of deformity of the lower limb in adults who have osteogenesis imperfecta. J Bone Joint Surg Am.

[19] Saldanha  KA, Saleh  M (2004). Limb lengthening and correction of deformity in the lower limbs of children with osteogenesis imperfecta. J Bone Joint Surg Br.

[20] Vlad  C, Georgescu  I (2012). Burnei’s procedure in the treatment of long bone pseudarthrosis in patients having osteogenesis imperfecta or congenital pseudarthrosis of tibia – preliminary report. Journal of Medicine and Life.

[21] Gross  RH (2012). The Use of Bone Grafts and Bone Graft Substitutes in Pediatric Orthopaedics: An Overview. J Pediatr Orthop.

[22] Firoozbakhsh  K, Moneim  MS (2004). Smart intramedullary rod for correction of pediatric bone deformity: A preliminary study. Clin Orthop Relat Res.

